# Supplementary material for: Are carbon emissions trading and green financial instruments synergistic? -Comprehensive quantitative research based on content analysis
Source: PLoS One. 2024 Mar 7;19(3):e0298601. doi: 10.1371/journal.pone.0298601 (PMC10919601; doi:10.1371/journal.pone.0298601)
Supplement: S1 Data — (ZIP) [file pone.0298601.s001.zip › Data/地级市Z_F工作报告.zip!/╝¬┴╓/┴╔╘┤╩╨/└┤╫╘╣1⁄2╓┌║┼í╛┬φ┐╦╩2╛▌═°í┐_2016.docx]

各位代表：

　　现在，我代表市政府，向大会报告政府工作。请予审议，并请市政协各位委员提出意见。

　　一、2015年工作回顾

　　刚刚过去的2015年，是辽源转型发展进程中很不平凡的一年。在省委、省政府和市委的坚强领导下，市政府同全市各族人民一道，积极应对新常态、新挑战，抓住战略机遇，奋力攻坚克难，经济社会发展呈现新局面。预计地区生产总值实现750亿元，增长7%；地方级财政收入实现28.1亿元，增长4.8%；全社会固定资产投资完成613亿元，增长13%；社会消费品零售总额达到205亿元，增长8.4%；外贸进出口总额达到2.7亿美元，增长10.2%；城乡常住居民人均可支配收入达到27433元和11025元，分别增长4.5%和5%。主要指标增幅处于全省上游，相关社会发展和节能减排指标完成年计划。

　　经济增长势头不减。全力克服经济下行压力，引增量、带存量、扩总量，保持经济增长处于合理区间。投资拉动、项目带动作用增强。在建亿元以上项目199个，其中10亿元以上项目39个。利源精制轨道车体材料6条生产线、麦达斯轻合金冷轧机和拉弯矫直机生产线、方大锻造8000吨锻压生产线、均胜汽车注塑成型和喷涂产品等49个项目投达产；金翼蛋品循环经济产业园、中农农副产品批发市场等项目进展迅速；凯旋王国创意园等项目恢复续建。投资对经济增长贡献率达到45%。工业经济逆势上扬。袜业、铝业、汽车零部件等接续替代产业支撑作用明显，重点企业发展势头强劲。实施工业技改项目207个，投资增长13.5%。产值超亿元企业228户，其中超10亿元企业达到35户。预计规上工业产值、增加值达到1443亿元和442亿元，分别增长9.3%和9%，增速列全省第二位，工业拉动经济增长5个百分点。有效需求不断释放。汽车消费和居民健康消费、网上消费持续升温，房产交易量有所回升。举办首届辽源冰雪旅游节和魁星文化庙会暨上海台商庙会，鴜鹭湖“花海”、东丰油菜花观赏区成为热点景区，全市旅游总收入增长25%以上。对外贸易拓展到50多个国家和地区，形成了以铝型材、袜品、农产品为骨干的出口产品格局。

　　结构调整取得实效。坚持多措并举、多点发力，促进经济结构稳中向好、好中定型。工业结构进一步优化。主导产业、特色优势产业产值分别增长11.3%和16.7%，占全市工业70%以上。以钢铁、煤炭、水泥等为代表的传统产业比重下降3.4个百分点。锂电池、锂电隔膜等战略性新兴产业不断壮大，竞争优势进一步巩固。农业现代化水平得到提升。克服干旱等不利因素，粮食产量达到44.7亿斤，高标准农田达到68万亩。棚室蔬菜发展到2.6万亩，食用菌产量突破7500吨。农产品加工业销售收入增长13.6%。东辽黑猪成为国家地理标志保护产品。东辽县被农业部确定为现代农业示范县。东辽河源头现代生态农业示范区项目列入中德政府合作签约计划。在全省县域综合考评中，东丰县位列第八位，东辽县列一类县第二十四位。服务业发展持续向好。把服务业作为吸引社会投资的重点，实施82个亿元以上项目。现代服务业不断壮大，支撑服务业占比提高1个百分点。以“智慧城市”承载和助力“互联网+”，辽源电子商务园区被确定为首批省级电子商务示范基地，我市荣获2015中国“智慧城市”最具投资价值潜力奖。科技创新步伐明显加快。新增2个省级中试中心、2个省级企业技术中心，创新型科技企业占全省13.5%，发明专利授权创历史最好水平。新增吉林省著名商标5件、名牌产品13个，首次投产新产品110种，新产品产值率达到22%。创建国家可持续发展实验区得到科技部批复。东北袜业荣获吉林省质量奖。园区集聚效应日趋显现。辽源经济开发区在建亿元以上项目42个，连昌工业园、新材料产业园投入使用。启动城区老工业区搬迁改造和采沉区综合治理，工业新项目全部入园建设。东丰、东辽开发区连续被评为省级先进工业集中区。

　　发展活力不断增强。各领域改革取得新进展。深入实施简政放权、放管结合改革，建立并公布部门权力清单，实现非行政许可项目“零审批”。理顺市场监管体制，撤销83个市直非常设机构。深化商事制度改革，全面实行注册登记认缴和“五证合一”登记。完成市区分税制改革和不动产登记机构划并、职能整合，成立农村综合产权交易中心。推进城镇化和农村改革，寿山镇被列为全省扩权试点重点城镇，10个乡（镇）完成土地确权。加快金融创新步伐，组建东辽农商村镇银行，土地经营权抵押贷款、土地收益保证贷款在支农强农方面发挥重要作用。对外开放收到新成果。积极参与民企吉林行、央企走进吉林、东博会等经贸交流活动，有针对性地组织务实精准招商，新录入省调度系统招商项目83个，到位资金129亿元。莎普爱思强身药业、青岛昌盛日电光伏农业、天津力神与鸿图锂电资本合作、蓝天城少年儿童素质教育基地等62个亿元以上项目落地建设。

　　基础设施逐步完善。重点项目扎实推进。辽长铁路建成通车，伊开高速二期、集双高速辽源段开工，辽源机场项目前期工作启动。新建农村公路188公里，改造桥梁40座。完成大梨树河城防工程，东辽河干流防洪工程全线铺开，灯杆河、仙人河防洪工程扎实推进，中央水利投资续建项目取得突破。“气化辽源”工程实现与主干管道联通，新增居民天然气用户8700户。市政建设任务全面完成。“九路五带两园”工程提前完工，实际改造街路27条，更新硬覆盖4万平方米，理石提档1.5万平方米。改造老旧供热管网42公里。启动南部新城地下综合管廊建设。新建改造12处景观带、4个主题花园，城区绿化覆盖率达到40%。升级改造垃圾中转站25座，环卫机械化清扫率提高到40.5%。乡（镇）承载能力不断提升。东丰县被列为5个省级生态城镇化试点之一，金州、寿山、渭津、安恕、那丹伯、拉拉河等重点镇建设加快推进。乡（镇）基础设施趋于完善，集中供热乡（镇）达到16个，有排水设施乡（镇）达到12个，有垃圾处理设施乡（镇）达到27个。生态环保工作得到加强。强力组织推进水源地综合治理，加快杨木水库湿地建设。国家矿山湿地公园主入口广场、矿山博物馆完工，恢复绿地41公顷。实施36个减排项目，单位工业能耗下降4.2%。天楹垃圾发电项目投入运营，城市垃圾无害化处理率达100%。清收林地5507公顷，植树造林148万株。40个农村连片整治项目全部完成。

　　民生状况显著改善。年度惠民实事计划全面完成，投入资金超过40亿元。居民收入稳步增长。新增城镇就业4万人，扶持创业7000人，组织农民外出务工17.5万人。建立健全企业工资集体协商和行业协商制度，连续11年实行养老金调待。完成机关事业单位工资调整，落实乡（镇）机关事业单位工作补贴。再次提高城乡低保标准，建立临时救助制度，惠及困难群众4.9万人。人居环境不断改善。新建和回购保障房9128套、64.1万平方米，回迁安置居民1万户。改造“暖房子”51.8万平方米，整治小区道路4.2万平方米。城市24小时实际供水率达到90%，解决3.26万农村居民饮水安全问题。收回公交线路9条，公营车辆比例达到62.4%。新增停车泊位2800个。道路交通和市容环境百日整治行动取得积极成效。各项社会事业协调发展。推进基础教育“全面改薄计划”、学校标准化建设和优秀教师校际交流，两县被评为“全国义务教育发展基本均衡县”，我市被教育部确定为“现代学徒试点市”。在全省率先启动城镇居民大病医疗保险，最高报销比例达到80%。组织国内外专家来辽诊治3200例，城市公立医院药价平均下降21%。建成和改造侵华日军辽源高级战俘营旧址展览馆等一批文化设施，各类文化场馆免费服务群众超过20万人次。新建改建养老机构6个，新增床位1238张，在全省首推医养融合养老服务模式。食品药品可追溯系统初步建立，食品检验检测中心基本完工。市民卡公交、缴费、消费等功能相继开通。

　　和谐局面持续巩固。依法治市工作深入开展。坚持依法行政，组建市政府法律顾问团，健全重大决策合法性审查和社会稳定风险评估机制。促进司法体制改革，深入开展“六五”普法，完善了法律援助等一系列制度，东丰县被评为全国法治创建先进县。探索多元化社会矛盾调解模式，依法妥善解决一批信访突出问题。平安辽源建设取得实效。完善立体化安防体系，八类严暴案件、命案、“两抢”案件同比分别下降19.3%、14.2%和19.6%，群众安全感满意度继续保持全省领先位置。我市被团中央命名为全国青少年安全教育基地，连续5年获得全国见义勇为城市奖。安全生产工作再创佳绩。开展“春季行动”“夏季攻坚”“秋冬会战”等专项整治，各类事故同比下降34.9%，道路交通事故死亡率下降22%。危化品领域连续9年未发生生产死亡事故。实现连续31年无重大森林火灾。我市连续7年获得省政府安全生产考核一等奖。协调推进民族宗教、外事侨务、广播电视、新闻出版、方志、档案、气象、地震、人防等工作和妇女、儿童、老龄、残疾人等各项事业。援疆工作深入推进。双拥共建和军民融合发展基础更加牢固。

　　政府自身建设得到加强。坚持依法科学民主决策，自觉接受人大法律监督、工作监督和政协民主监督，充分听取社情民意，办结代表建议63件、政协提案90件。扎实开展“三严三实”专题教育，收集汇总、列入清单的11个方面问题正在落实整改。注重提高行政效能和降低行政成本，政府会议、文件数量分别下降22%和17%，“三公”经费下降10%左右。强化纪律和规矩意识，严格落实党风廉政建设“两个责任”和“一岗双责”，纪检监察机关立案396件、查处415人。

　　过去的一年，我们遇到的困难超出预期，取得的成绩更显来之不易。这是市委坚强领导的结果，是市人大、市政协和市级各民主党派、工商联、人民团体、无党派人士鼎力支持的结果，是全市各县区、各部门和广大干部群众拼搏实干的结果，是情系家乡发展的老领导、老同志和驻辽部队、中省直单位及海内外朋友热情帮助的结果。各个方面付出了艰辛努力，凝聚起了辽源转型发展的强大正能量。在这里，我代表市政府向大家表示崇高的敬意和衷心的感谢！

　　各位代表，随着2015年工作结束，“十二五”也实现了胜利收官。过去的5年，辽源各项事业协调健康发展，特别是形成了新的接续替代产业架构，解决了一系列历史遗留问题，开启了转型升级、创新发展新局面。全市地区生产总值、规上工业总产值、固定资产投资、地方级财政收入年均分别增长9.8%、19.5%、15.7%和10.5%。城乡面貌发生深刻变化，建成了一批交通、能源、水利重大项目和市政重点工程，常住人口城镇化率达到54.5%。改革开放不断深化，大量制约转型发展的体制机制障碍被清除，大批外来投资项目在辽源落地生根。人民福祉持续增进，就业、社保、教育、医疗、文化等公共服务得到改善，5万余户棚户区、采沉区居民喜迁新居。社会治理能力进一步提升，和谐稳定局面不断巩固。生态文明建设扎实推进，节能减排和污染治理取得积极成效。政治生态呈现新气象，广大干部群众万众一心、干事创业，走出了一条具有辽源特色的转型发展新路。所有这些，为“十三五”发展和全面建成小康社会打下了坚实基础，坚定了我们砥砺奋进的信心和决心。

　　在总结成绩和经验、充分认识下步发展战略机遇和有利条件的同时，我们清醒地看到转型发展中的矛盾和政府工作中的问题。从宏观层面看，经济增速换档、结构调整阵痛、动能转换困难相互交织，经济下行压力仍然较大。就我市而言，主导特色产业总量不够大，结构性矛盾比较突出，发展的内生动力和活力还没有充分释放；要素成本、融资瓶颈、生态环境容量等因素在一定程度上制约了重大项目落地；政府债务负担较重，财力状况与刚性支出需求不匹配；安全发展的基础还不够牢固，影响社会稳定的因素依然存在；政府的创新意识、法治观念、服务效率有待进一步提升。这些问题既是今后改进工作的重要方向，也是当前迫切需要解决的重点环节。

　　二、“十三五”经济社会发展的总体构想

　　“十三五”时期，是辽源加快转型升级的关键时期，是全面建成小康社会的决胜阶段。描绘和实施好未来5年蓝图，对于推动全市经济社会持续健康发展意义重大。

　　提交本次会议讨论审议的《辽源市国民经济和社会发展第十三个五年规划纲要（草案）》[以下简称《纲要（草案）》]，是在市委高度重视和统一领导下完成的。编制工作历时18个月，期间起草组深入研判形势、分析市情，积极主动与省“十三五”规划有效对接，并借助了国内专家力量，广泛征求、认真吸纳了社会各界的意见建议。《纲要（草案）》全面贯彻了新一轮东北振兴的战略部署和市委六届五次全会通过的“十三五”《规划建议》，是上情下情融会贯通、各个方面群策群力的智慧结晶，更是指导辽源未来5年发展的重要遵循。

　　《纲要（草案）》提出，“十三五”时期，要以转型升级为主线，以如期建成小康社会为目标，经济保持中高速增长，统筹城乡发展成效明显，人民生活水平全面提高，体制机制创新步伐加快，社会文明程度显著提升，生态文明建设扎实推进，努力把辽源建成国家资源型城市转型示范市。《纲要（草案）》有关目标体系的确定，经过了反复测算和研究论证，符合我市现阶段实际和未来发展预期，是积极稳妥、经过努力可以完成的。组织实施中将突出以下发展理念和工作重点：

　　一是注重创新发展，致力转型升级。加快由要素驱动向创新驱动转变，做好有中生新、无中生有两篇文章，装备制造和农产品深加工产业达到千亿级规模，高精铝、纺织袜业、医药健康、蛋品加工、新能源产业分别打造两个200亿级、三个100亿级产业集群。二是注重协调发展，促进区域统筹。坚持全域规划、科学布局，实施主体功能区划，完善重大基础设施，尽快培育优势、补齐短板，推动辽白经济一体化建设，实现三次产业、区域城乡、经济社会协调并进。三是注重绿色发展，巩固生态优势。秉承“绿水青山就是金山银山”，坚持保护与开发并重，严守生态红线，全力保护好我们的母亲河，争创全国“循环经济试点市”，建设吉林省中南部绿色屏障区。四是注重开放发展，拓展发展空间。主动融入国家和我省新一轮高水平对外开放布局，深化同东北亚地区、“一带一路”沿线国家和欧盟成员国合作，积极推动面向长春和环渤海地区的经济协作，打造发达经济区产业转移承接地和特色优势产业集聚区。五是注重共享发展，增进群众福祉。坚持以百姓需求为导向，增加公共服务和公共产品供给，确保完成脱贫攻坚任务，办实办成一大批民生实事，让辽源人民有更多获得感和幸福感。

　　三、2016年工作安排

　　2016年，市政府将认真落实省委、省政府和市委的决策部署，科学认识、主动适应、积极引领发展新常态，以加快转型升级为主线，以重大项目建设为抓手，以体制机制创新为动力，统筹做好稳增长、调结构、促改革、惠民生、保稳定等重点工作，努力实现“十三五”开好局、起好步。主要预期目标是地区生产总值增长7%，地方级财政收入增长6%左右，固定资产投资增长11%以上，社会消费品零售总额增长8%左右，居民消费价格指数涨幅3%，城乡常住居民人均可支配收入与经济增长同步，城镇登记失业率控制在3.9%以内，必保完成节能减排计划。

　　（一）全力保持经济稳定增长。把稳增长作为经济工作的重中之重，改善有效供给，扩大有效需求，注重提高经济运行质量和效益。着力稳定工业经济增长。千方百计提高要素配置效率，加快在建项目投产达效，支持重点企业开足马力生产和扩大再生产。更多关注立市型产业和成长型企业，实施“中小企业入规升级”专项行动和“小微企业培育”工程，新增规模以上企业30户、小微企业400户。坚持分类分企指导，精准研究企业脱困措施，努力解决流动资金、技术改造、资产重组、市场营销等方面的实际问题。全力争取省精准调控资金，最大限度发挥各类专项资金的使用效率和引领作用，坚决防止资金沉淀和“体外循环”。着力发挥投资关键作用。实施亿元以上项目180个，其中10亿元以上项目37个。重点推进东辽河源头现代生态农业示范区、麦达斯铝业2.35万吨挤压生产线等项目。跟进国家新一轮东北振兴政策，组织申报200个以上重大项目，力争一批重大交通、水利、能源和城市基础设施项目列入开工计划。争取国家专项支持，引导民间资本、外来投资参与，扎实推动城区老工业区搬迁改造和采沉区综合治理。滚动谋划200个以上重大项目，充实“十三五”项目储备库，精心做好前期工作，提高项目成熟度和成功率。着力促进消费升级。引导住房消费、汽车消费，鼓励发展新型商业，培育健康、养老、文化、旅游等消费热点，充分释放农村消费巨大潜力，不断满足个性化、多样化消费需求。引进大型商业企业，探索设立高端商品直销中心，吸引域外消费回流。加快建设城市商业副中心，开展特色化促销活动，发展服务本地、辐射周边、线上线下交汇的农副产品、纺织品、建材等专业化大市场。着力推进供给侧结构性改革。支持煤炭、钢铁、水泥、酒精等行业重点企业参与产能合作、兼并重组、实施技改项目，鼓励矿山装备、建筑机械制造行业转产新产品，积极稳妥地处置一批“僵尸企业”。推动房地产“去库存”，把回购存量商品房作为棚改安置房重要来源，鼓励农民工、外来人口在城市购房，根据去库存进度调节土地供应。开展降本增效专项行动，实行收费清单管理模式，有效降低实体经济的制度性交易成本、人工成本、电力价格和物流成本、财务成本。增加优质供给，努力在补齐补强服务业、基础设施、人力资本等短板方面实现突破。

　　（二）积极培育转型升级新优势。加快新旧动力转换，致力构建现代产业体系。深入实施创新驱动战略。全面推进国家可持续发展实验区建设，完善产学研用协同创新体系，建设吉林科技大市场辽源分市场。推进医药高新技术特色产业基地和针纺联盟建设，争取省铝合金及深加工高新技术特色产业基地落地辽源。出台促进大众创业、万众创新的配套政策，培育创客空间、创新工场10个以上。实施环东北人才集聚战略，注重企业家队伍建设，推广现代学徒制度，充实高中端人才储备库。统筹规划辽源经济开发区与东辽开发区、西安工业集中区产业布局，加快辽白经济一体化进程。启动东北袜业第二园区和欧亚北区域、龙山现代服务业新型综合城区、西安区创新转型发展示范区建设。加快产业结构调整步伐。装备制造业突出“提升”，推动由零件加工向部件生产、由部件生产向整机制造升级，计划实施57个技改项目。农产品深加工业突出“延伸”，支持重点企业发展循环经济，提高精深加工能力和辐射带动作用，计划实施20个重点项目。高精铝加工业突出“精制”，全方位发展挤压、热轧、熔铸、焊接、铝箔工艺，开发产业链下游高端产品，吸引同类企业配套，巩固行业领先地位。纺织袜业突出“品牌”，承接产业转移与产品更新换代升级并重，加快形成产品、技术、装备、市场全产业链模式，推动全国棉袜产业知名品牌示范区建设，争取袜制品原材料国检中心投入使用。医药健康产业突出“优化”，加强与辅仁药业、莎普爱思、修正药业、九州通医药等大集团合作，支持梅花鹿产品深度开发。蛋品加工业突出“循环”，推进金翼蛋品后续深加工项目，做大做强循环经济产业园，全力打造国内最大的蛋品深加工企业集团。新能源产业突出“扩张”，支持锂离子电池、高性能隔膜纸生产企业扩大规模，积极推进光伏发电、风气互补发电、生物质能源利用等重点项目，以科技突破带动产业升级。大力推动服务业增量提质。生产性服务业突出“配套”，加快发展第三方物流、融资租赁、信息、电子商务、节能环保等新业态，建设开发区现代物流港，完善城市共同配套体系，助力产业发展向价值链高端延伸。生活性服务业突出“多元”，促进商贸、餐饮、家政、健康、教育、养老等产业加快发展，搞好夫余国后期王城等特色历史文化资源保护开发，打造凯旋王国创意园等新的旅游热点。

　　（三）加快农业现代化建设。推动农村一二三产业渗透融合，促进农业现代化与新型城镇化相衔接，提高农业质量、效益和竞争力。大力发展种养加特色农业。稳定耕地作物面积，加强农田水利基本建设，调动农民种粮积极性，实现粮食稳产增产。鼓励应用智能温室、物联网等先进技术，加快建设一批特色种植园、采摘园和高标准生态休闲农庄。实施“健康有机米”“放心肉蛋奶”和“果菌蔬蚕”增量创收工程，着力打造粮食精深加工、畜牧业循环经济、特色农产品三大集群。加快吉东种业改制重组和育繁推一体化进程。统筹重点镇和新农村建设。全域规划新型城镇化布局，完善14个特色小城镇基础功能，推进重点镇基本建设。引导乡村企业向园区集中、空心村向中心村集中，促进土地、技术、劳动力等要素在城乡间合理配置，不断夯实新型城镇化的产业基础。更加注重以人为核心，深入实施农村环境综合整治，加快基本公共服务向乡镇、村屯延伸，推进新型农村社区建设，争取再建设一批美丽乡村。促进农民持续增收。推进适度规模经营和土地林地承包流转，开发农民外出务工就业的机会和岗位，增加农民的工资性收入和财产性收入。支持城市资本和服务下乡，培育农村电商、农产品定制等“互联网+”新业态，加快构建农业生产性服务业体系。引导大中专毕业生、返乡人员领办合作社和家庭农场，支持龙头企业通过直接投资、参股经营、签订长期合同等方式参与农村建设，打造一批袜子村、剪纸村、农民画村和梅花鹿、食用菌等专业村。

　　（四）扎实提升城市规划、建设、管理水平。围绕实施主体功能区划、推进市县“多规合一”、提升中心城区功能，开展城市总体规划修编，完成城市风貌、工业园区、海绵城市等专项规划。启动城市建设管理提挡升级攻坚计划，强力推动基础设施建设，显著改善城市面貌和人居环境。重点实施10项建设工程：一是重点交通工程，按期组织伊开高速二期和集双高速公路建设，加快机场、铁路项目前期工作，规划启动城市“大三环”建设。二是重点水利工程，加速东辽河城防工程和渭津河、灯杆河、仙人河治理，争取“引松入辽”二期辽源支线开工建设。三是“气化辽源”覆盖工程，新增居民天然气用户5000户，逐步向企业提供稳定气源。四是市政“十路一园”工程，实施人民大街畅通改造和福镇大路东出口综合改造，完成30条巷路改造。五是管网管廊建设工程，改造给水、雨水和污水管网20公里，建成南部新城地下综合管廊8公里，启动东辽河、仙人河两岸管廊建设工程。六是城市危楼改造工程，有计划地拆除、改造、维修加固一批旧危楼房，确保群众居住安全。七是国家级卫生城、园林城、森林城创建工程，统筹推进软硬件建设，争取国家级园林城达到申报条件。八是城市“亮化”工程，市区街路、广场、沿河景观带、高层建筑、标志性建筑基本实现亮化。九是南部新城续建工程，完成工商学校基本建设收尾和横七路项目，抓好辽源职业技术学院迁建前期工作。十是“智慧城市”应用项目工程，务实推进与深圳中兴通讯、吉林联通的战略合作，启动城市运营中心和30个智慧社区建设，开展物流寄递业和无线接入热点安全管理系统建设。重点开展10项专项整治：一是整治城市交通秩序，纠治非法营运、违章行车停车、占用消防通道以及车窗抛物等不文明行为。二是整治“马路市场”，合理规划设置农贸市场及早市、夜市，禁止在城市主干路摆摊设点。三是整治“马路工厂”，清理沿街修车、加工商户，实现常态化管理。四是整治餐饮油烟污染和露天明火烧烤，规范夏季大排档。五是整治违法建筑，严禁各类私搭乱建行为，开展全市范围违法建筑拆除行动。六是整治损毁市政设施，及时发现、严厉打击各种破坏行为。七是整治城区河道卫生，合理设置垃圾存放点和中转站，严禁向河道倾倒生活垃圾。八是整治建筑扬尘和渣土运输车辆，严格规范建筑工地施工，特别是严禁在主要街区、居民区野蛮施工。九是整治侵占公共用地行为，重点清收被圈占的城市和住宅小区公共绿地，尽快恢复原有功能。十是整治城乡结合部卫生，坚持属地管辖、权责明确，实现制度化管理和常态化保洁。

　　（五）进一步深化改革扩大开放。认真贯彻落实顶层设计，更加注重体制机制创新。深化行政体制改革。完善部门权力清单，建立责任清单、投资负面清单和财政资金管理清单。建设集权力运行、政务服务、效能监察为一体的行政权力运行平台，着力打通服务群众和企业的“最后一公里”。深化经济领域改革。进一步完善商事制度和市场监管改革后续机制，清理并废除对民营经济的歧视性政策。积极承接辽矿集团剥离企业办社会职能，推进公交公司改革，妥善解决厂办大集体等国企改制遗留问题。深化财税机制改革。推进建立现代财政制度，完善政府债权追偿机制和政府债务风险防控机制。完成公务用车改革，全面开展不动产登记。扩大政府购买服务范围，鼓励社会资本参与政府投资项目。深化金融改革创新。改进国有担保公司经营模式，增强政府融资平台担保能力。抓住股票发行注册制度改革契机，推动有条件企业上市融资。完善金融生态环境建设相关制度，建立清偿银行债权与扩大信贷投放挂钩机制。深化农村改革。完成东辽县农村土地承包经营权确权登记颁证，开展农业经营性建设用地入市、宅基地制度改革试点。探索建立农业生态产业引导基金。继续深化社会事业领域和生态文明体制改革。

　　坚定不移地推进双向开放，形成开放发展新局面。积极开展精准招商。以行业领军骨干企业为主体，推动与域外大企业合作项目加快建设，吸引企业协作，延伸产业链条，促进集群化发展。利用第三届辽源国际袜交会、台资企业吉林行、世界500强走进吉林等活动平台，务实对接和签约落地一批新项目、大项目，力争引进域外资金600亿元，实际利用外资3.3亿美元。支持企业和产品走出去。引导本地企业靠大联强、开展并购，引进先进技术、优质资源。组织本地产品走出去，把辽源的品牌宣传出去，借时借力造势，开拓国内、国际两个市场，争取共鸣共振共赢效果。发挥铝业、袜业、农产品出口基地作用，提高外贸进出口对经济增长贡献率。努力形成融合发展格局。围绕我省中部创新转型核心区建设，在吉林向南开放和打造辽源、四平、铁岭、通辽经济协作区方面发挥积极作用。发展与一汽、长客、吉化等大型企业相关的配套产业，加快融入全省产业战略布局。

　　（六）切实保障和改善民生。坚持保基本、兜底线、促公平原则，继续组织实施惠民实事计划。抓好基本民生。支持灵活就业和新就业形态，解决好高校毕业生、退伍军人、“4050”人员和残疾人就业问题，力争城镇新增就业4万人，扶持创业7000人。做好普及高中教育相关工作，促进公办教育与民办教育协调发展。培育专科医院和全科医生，推广公建民营、医养融合养老模式。筑牢社保底线。推进机关事业单位与企业养老保险并轨，提高养老金待遇水平，落实失业金“援企稳岗”政策。保持城乡低保标准与社会平均工资增长同步。健全临时救助、慈善捐助、大病医保等配套政策，加强下岗职工、失地农民、农村五保户、低保边缘户、残疾人的基本生活保障，切实解决空巢老人、留守儿童、失独家庭遇到的具体困难。落实“全面二孩”政策。解决民生热点问题。新建保障房8321套、56万平方米，安置居民7000户，基本完成“暖房子”改造。加强小区物业管理，完善居民生活服务设施。解决农村居民饮水安全问题7400人。扩大市民卡、健康卡应用范围，提高社会事业领域智能化服务水平。积极促进文化惠民、体育强民，建设南部新城多功能全民健身中心。突出抓好脱贫攻坚。严格落实脱贫攻坚时间表和各层级责任制，建立健全脱贫验收和退出机制，按照“一确保、两不愁、三保障”目标，在产业扶贫、开发扶贫、政策扶贫上精准发力，力争年内1万贫困人口实现脱贫。

　　（七）着力加强生态文明建设。坚定不移地走绿色低碳循环发展之路，构建绿色产业体系和空间格局，促进人与自然和谐共生。做好构筑绿色生态屏障的“加法”。严守生态保护红线，继续开展植树造林、封山育林、退耕还林。加快推进鴜鹭湖国家湿地公园、国家矿山湿地公园、寒葱顶国家森林公园建设。做好资源节约集约利用的“减法”。实行最严格的节约集约用地制度，开展违法用地、闲置用地专项整治。实行最严格的水资源管理制度，更新改造节水工艺，推广普及节水产品。实行能源消耗总量和强度控制，支持重点领域、重点企业节能改造。做好发展低碳循环经济的“乘法”。围绕创建国家级“循环经济试点市”，鼓励企业修旧利废，抓好农村秸杆、畜禽粪便综合利用。启动垃圾焚烧发电二期工程，逐步完善农村垃圾“村屯收集、乡镇运输、市县处理”机制，促进垃圾的减量化、无害化、资源化。做好水、大气、土壤污染防治的“除法”。实施杨木水库水源地一级保护区隔离工程，严禁东辽河流域企业和生活污水直排。强化工业废气粉尘、秸秆焚烧、汽车尾气、燃煤小锅炉等治理，尽最大努力改善区域空气质量。推进工矿废弃土地复垦和植被恢复，深入实施黑土地保护工程。

　　（八）加快推进依法治市进程。坚持系统治理、综合治理、源头治理与依法治理有机统一，积极营造遇事找法、办事依法、解决问题用法、化解矛盾靠法的法治环境。加强社会治安综合治理和风险管控，严密防范和有力打击各种违法犯罪，进一步提高群众安全感。加强法律服务中心、法律援助中心建设，完善涉法涉诉信访依法终结制度。健全完善安全生产“党政同责、一岗双责、失职追责、齐抓共管”责任体系，构建“依法治安”监管防控体系，持续开展安全隐患排查治理，坚决遏制较大以上事故。搞好防汛抗旱、防灾减灾等工作，提高应急反应能力。着力加强食品药品监管，让损害人民生命健康行为付出沉重代价。加强全民国防动员和国防后备力量建设，适时做好驻辽部队改革的服务保障，促进军民融合深度发展。

　　各位代表，为完成新一年的目标任务，市政府将进一步把加强自身建设牢牢抓在手上，拿出务实管用的具体措施，履行好基本职责，在创建人民满意政府上迈出新步伐。要坚持依法行政。所有政府工作人员都要自觉尊法学法守法用法，所有政府行为都要置于法律规范约束之下。完善依法科学民主决策制度，建立重大国有资产处置决策机构，实行决策终身责任追究制度和倒查机制。要强化从严治政。把政府作风建设和反腐倡廉建设摆到更加突出位置，着力整治不作为、乱作为问题。加强腐败风险管控，实现公共资金、重大工程、国有资产、国有资源等重点领域审计、监察全覆盖。要优化政府服务。主动接受人大、政协和社会各界监督，探索建立公民、法人评价制度，对大家反映的问题即知即改，以社会满意程度倒逼政府服务升级。进一步改进审批管理机制，扩大网上审批比重，建立市长公开电话、市长信箱与各行业各部门热线电话的联动机制，及时回应解决群众的合理诉求。要狠抓工作落实。大力营造敢于担当、善于担当、体现担当的浓厚氛围，完善绩效管理和考核评价体系，强化督查检查、跟踪整改和相应的奖惩机制，以落实效果检验政府执行力和公信力。

　　各位代表，辽源已经站在新的历史起点。唯有一往无前才能扛起光荣使命，唯有拼搏实干才能实现宏伟蓝图。让我们紧密团结在以习近平同志为总书记的党中央周围，在市委的坚强领导下，增强发展自信，锐意开拓进取，为辽源人民生活得更加美好而努力奋斗！
